# Recent Advances in Visible-Light Photoredox Catalysis for the Thiol-Ene/Yne Reactions

**DOI:** 10.3390/molecules27030619

**Published:** 2022-01-18

**Authors:** Qian Xiao, Qing-Xiao Tong, Jian-Ji Zhong

**Affiliations:** 1 School of Chemistry and Environmental Engineering, Hanshan Normal University, Chaozhou 521041, China; 19qxiao@stu.edu.cn; 2 Key Laboratory for Preparation and Application of Ordered Structural Materials of Guangdong Province, Department of Chemistry, Shantou University, Shantou 515063, China; 3 The Chemistry and Chemical Engineering Laboratory of Guangdong Province, Shantou 515063, China

**Keywords:** photoredox catalysis, thiol-ene/yne reaction, synthetic methodologies, green chemistry

## Abstract

Visible-light photoredox catalysis has been established as a popular and powerful tool for organic transformations owing to its inherent characterization of environmental friendliness and sustainability in the past decades. The thiol-ene/yne reactions, the direct hydrothiolation of alkenes/alkynes with thiols, represents one of the most efficient and atom-economic approaches for the carbon-sulfur bonds construction. In traditional methodologies, harsh conditions such as stoichiometric reagents or a specialized UV photo-apparatus were necessary suffering from various disadvantages. In particular, visible-light photoredox catalysis has also been demonstrated to be a greener and milder protocol for the thiol-ene/yne reactions in recent years. Additionally, unprecedented advancements have been achieved in this area during the past decade. In this review, we will summarize the recent advances in visible-light photoredox catalyzed thiol-ene/yne reactions from 2015 to 2021. Synthetic strategies, substrate scope, and proposed reaction pathways are mainly discussed.

## 1. Introduction

**Figure 1:**
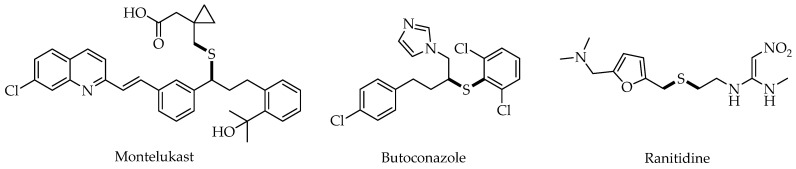
Selected pharmaceuticals bearing thioether moiety.

**Scheme 1:**
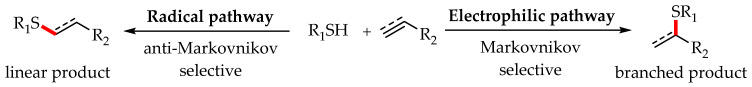
The general reaction pathway for the thiol-ene/yne reactions.

Thioethers, as a large and important member of organosulfur compounds, are extensively present in natural products and pharmaceuticals [1,2,3,4] (Figure 1). For instance, Montelukast is a potent and selective inhibitor, exhibits outstanding effect in the prophylaxis and chronic treatment of asthma [5,6]. Butoconazole, an antifungal agent, is active against many dermatophytes, such as vulvovaginal infections caused by candida albicans [7,8]. Ranitidine, a well-known drug which is widely used in the treatment of duodenal and gastric ulceration, also contains the typical carbon-sulfur bond [9]. Moreover, thioethers are valuable synthetic intermediates and important building blocks to access more functionalized sulfur-containing molecules such as sulfoxides and sulphones, consequently, they play an important role in synthetic chemistry and material science [10,11,12,13,14]. Due to the importance of thioethers, the development of methodologies for the synthesis of thioethers are of particular significance, and tremendous efforts have been made. In this regard, the thiol-ene/yne reaction, the direct hydrothiolation of alkenes/alkynes with thiols, represents one of the most powerful and atom-economic approaches for the construction of carbon-sulfur bonds, and has attracted much attention [15,16,17,18,19]. Generally, this simple reaction could proceed via an electrophilic pathway or a radical pathway, leading to two different types of thioethers, the branched product via Markovnikov addition, and the linear product via anti-Markovnikov addition (Scheme 1). Due to the high reactivity of sulfur, in most cases, the reaction tends to proceed via a radical pathway to access the anti-Markovnikov type products traditionally initiated by thermal or UV-light activation, using a stoichiometric radical initiator or by direct UV-light irradiation [20,21,22,23]. These traditional methodologies often required either stoichiometric reagents or a specialized UV photo-apparatus, and suffered from the uncontrollable radical pathways, the formation of side-products, or an unsatisfactory reaction yields, which limited the further application of these hydrothiolation reactions. Therefore, the focus gradually moved to the development of milder and greener methodologies to accomplish this prototypical “click” reaction. Among them, visible-light photoredox catalysis has received substantial attention owing to its inherent characterization of environmental friendliness and sustainability [24,25,26,27,28,29,30,31,32,33,34,35]. During the last decades, tremendous research efforts have been devoted to the development of visible-light photoredox catalysis as a powerful tool for organic transformations, and it has also been demonstrated to be a reliable and efficient way for the thiol-ene/yne reactions to approach the carbon-sulfur bonds under mild conditions. For instance, in 2013, Yoon’s group reported a pioneering visible-light photocatalytic thiol-ene reaction using transition-metal photocatalyst instead of the use of UV irradiation under mild reaction conditions [36]. One year later, Stephenson’s group developed a similar mild and selective visible-light photocatalytic thiol-ene reaction to access the sulfur-containing compounds [37]. Since then, the visible-light photoredox catalytic thiol-ene/yne reactions has experienced a great development. Various photocatalytic thiol-ene/yne systems for the diverse carbon-sulfur bonds formation have been developed.

Despite the great achievements that have been made in this field, to the best of our knowledge, there are few comprehensive reviews devoted to this emerging topic. In this review, we will aim to summarize the recent advances in visible-light photoredox catalyzed thiol-ene/yne reactions from 2015 to 2021. The main content will be classified into two parts based on the selectivity of the reaction, including the anti-Markovnikov-selective reactions and the Markovnikov-selective reactions. Examples of the visible-light photocatalytic thiol-ene/yne reactions that were applied in the field of biology and materials will be ignored in this article; we will mainly focus our attention on the literatures involving the development of synthetic methodologies for thiol-ene/yne reactions. We hope that this review will be useful for the medicinal and synthetic organic chemists, especially the chemists who focus on organosulfur chemistry, and promote the development in this field.

## 2. Photoredox Catalytic Anti-Markovnikov-Selective Thiol-Ene/Yne Reactions

In most cases, due to the high reactivity of sulfur, the thiol-ene/yne reactions tend to proceed via a radical pathway to provide the anti-Markovnikov products by visible-light photoredox catalysis. Since Yoon and Stephenson’s pioneering works, various photocatalytic systems with anti-Markovnikov selectivity have been reported for synthesis of the diversity of organosulfur compounds.

### 2.1. Photocatalytic Anti-Markovnikov Thiol-Ene Reactions for Synthesis of Alkyl Thioethers

**Scheme 2:**
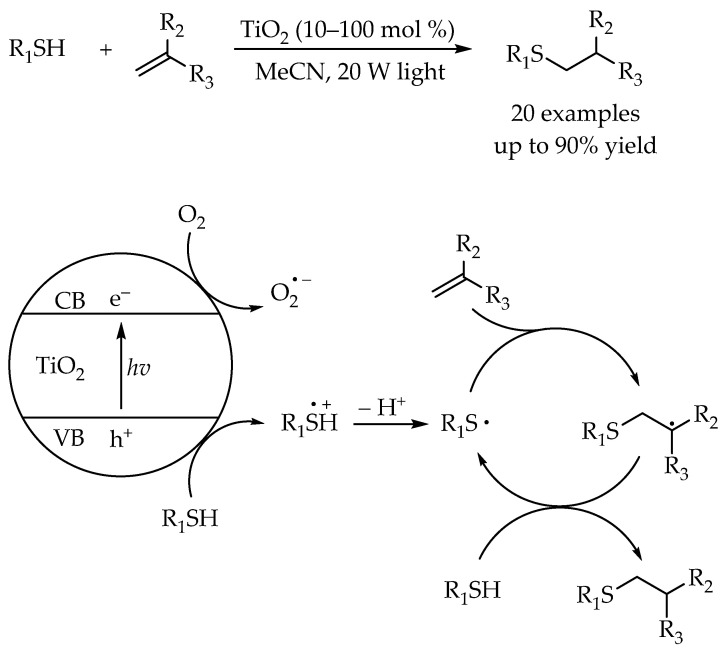
Visible-light-promoted thiol-ene reaction using titanium dioxide.

In 2015, Greaney’s group developed a visible-light-promoted thiol-ene reaction for synthesis of alkyl thioethers using TiO_2_ as photocatalyst [38]. Compared with the commonly used transition-metal-based photocatalysts, herein, TiO_2_ was cheap, readily accessible, and provided the advantage of catalyst recyclability. This work proved that besides the transition-metal complexes, inorganic semiconductors could also act as an efficient photocatalyst in the thiol-ene reaction. According to the proposed mechanism, the reaction was initiated by a photoinduced electron transfer between the thiol and the excited TiO_2_ to generate a thiyl radical. Subsequently, the anti-Markovnikov-selective addition of the thiyl radical to the alkene would provide an alkyl radical which could abstract hydrogen atom from an unreacted thiol to generate the corresponding alkyl sulfides (Scheme 2).

**Scheme 3:**
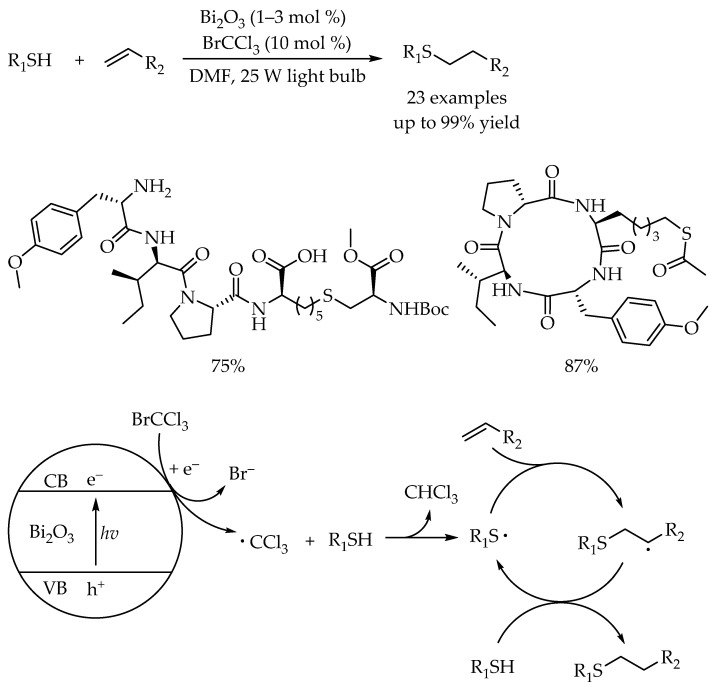
Visible-light-photocatalyzed radical thiol-ene reaction using bismuth oxide.

In the same year, Fadeyi’s group reported a visible-light photocatalytic anti-Markovnikov hydrothiolation reaction for alkyl sulfides synthesis using another nontoxic and inexpensive inorganic semiconductor Bi_2_O_3_, with broad functional group compatibility [39]. In contrast to Greaney’s work, a variety of complex biologically relevant scaffolds such as linear tetrapeptide or cyclic tetrapeptide could readily undergo late-stage functionalization in this protocol, demonstrating the practicability of the protocol. The plausible mechanism showed that the photoinduced single electron transfer (SET) between Bi_2_O_3_ and bromotrichloromethane (BrCCl_3_) would result in the formation of trichloromethyl radicals, which acted as a radical mediator to abstract a hydrogen atom from the thiol to generate a thiyl radical for inducing the radical thiol-ene reaction to access the corresponding anti-Markovnikov products (Scheme 3).

**Scheme 4:**
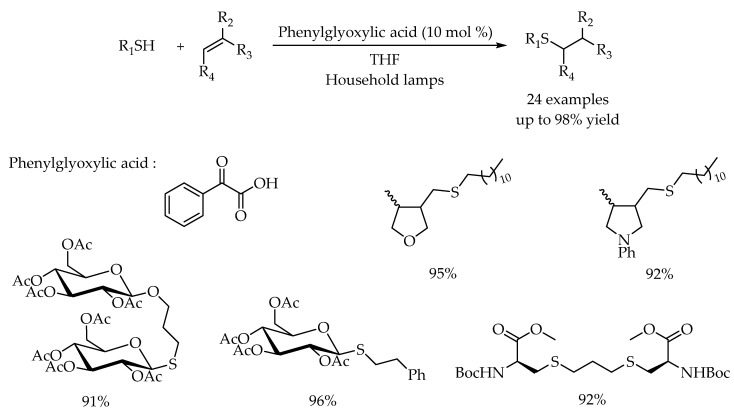
Visible-light-photocatalyzed thiol-ene reaction by phenylglyoxylic acid.

Transition-metal-based complexes were commonly employed as a catalyst in the photoredox catalyzed thiol-ene reactions. However, a problem that cannot be ignored is the intrinsic disadvantages of transition-metal complexes such as high cost, potential toxicity, and problematic removal of residual metal impurities. An alternative method was the introduction of inexpensive and less toxic metal-free organophotocatalyst. Therefore, Kokotos and co-workers reported a metal-free photoredox catalytic method for the radical thiol-ene reaction [40]. Phenylglyoxylic acid was proved to be the most efficient organophotocatalyst providing the corresponding anti-Markovnikov alkyl thioethers in good to excellent yields. Besides the simple olefins, dienes could also serve as compatible partners. When diallyl ether and *N*,*N*-diallylaniline were employed as the substrates, a radical cascade cyclization happened to provide the alkyl sulfides with a tetrahydrofuran or pyrrolidine moiety in 95% and 92% yields, respectively. Further experiments demonstrated that such a mild and green protocol could be successfully applied to the modification of peptide and glycoside, and they also showed broad functional group tolerance (Scheme 4).

**Scheme 5:**
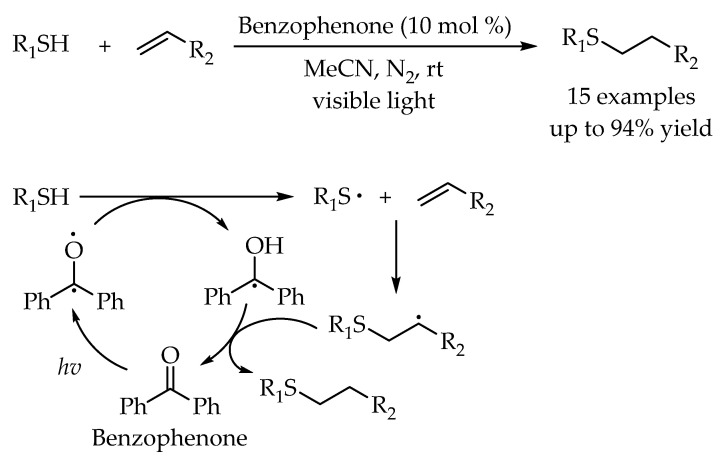
Visible-light-photocatalyzed thiol-ene reaction by benzophenone.

Furthermore, Singh and co-workers utilized benzophenone as an inexpensive organophotocatalyst to promote the carbon-sulfur bond formation through the reaction of thiols and olefins under visible-light irradiation [41]. Diverse anti-Markovnikov adducts of thiols and olefins were obtained in good to excellent yields. For the proposed reaction pathway, the interaction between the thiol and the excited-state benzophenone could generate a thiyl radical and the ketyl radical by a hydrogen atom transfer (HAT) process. Then, the thiyl radical addition to the olefins furnished a new alkyl radical, which could abstract a hydrogen atom from the ketyl radical to fulfill the photocatalytic cycle and afford the alkyl sulfides (Scheme 5).

**Scheme 6:**
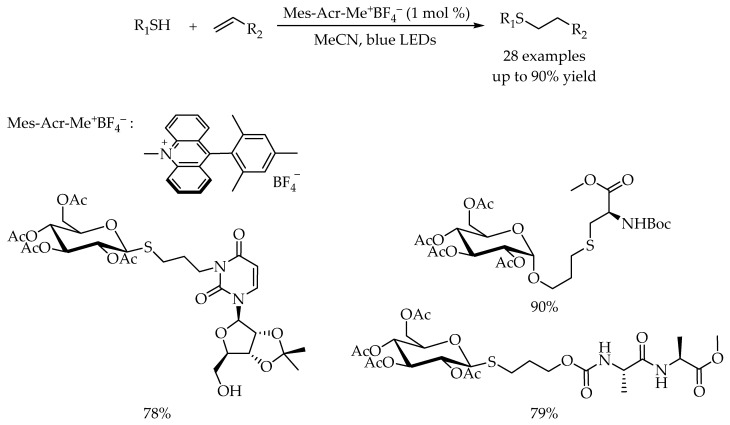
Visible-light-photocatalyzed thiol-ene reaction using 9-mesityl-10-methylacridinium tetrafluoroborate.

In 2017, Wang and co-workers reported a visible-light-promoted radical hydrothiolation of alkenes with thiols using 9-mesityl-10-methylacridinium tetrafluoroborate as photocatalyst [42]. The reaction proceeded smoothly with a variety of alkenes and thiols, giving the alkyl thioethers in excellent yields. Moreover, examples of the synthesis of glycoconjugates between glycosyl thiols and amino acid derivatives demonstrated the potential applicability of this strategy. Particularly, the coupling of glucose thiol with a nucleoside derivatives and dipeptide derivatives could afford the glyconucleoside and glycopeptide in good yields (Scheme 6). 

**Scheme 7:**
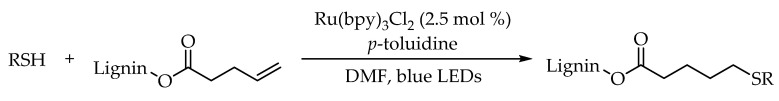
Visible-light-photocatalyzed thiol-ene reaction for modification of natural lignin.

Polymeric modification of lignin was necessary. For this target, Chung’s group developed a lignin-involved visible-light-photocatalyzed thiol-ene reaction using Ru(bpy)_3_Cl_2_ as a photocatalyst [43]. This low energy and environmentally friendly protocol was tolerant to a variety of functional groups, resulting in a high conversion of alkenes with reaction yields from 81% to 97%. Moreover, excellent reaction efficiency could also be achieved even after 4 h irradiation of natural sunlight (Scheme 7). This protocol provided a simple and efficient way for constructing the structural complexity of lignin containing sulfur functional groups.

**Scheme 8:**
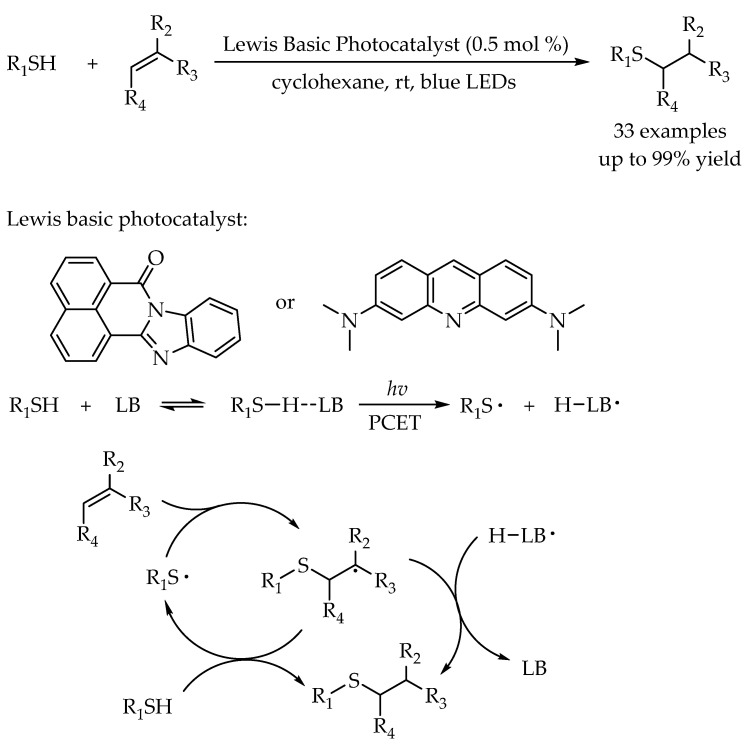
Visible-light-photocatalyzed thiol-ene reaction via a proton-coupled electron transfer process.

As mentioned above, in the radical thiol-ene reaction, a key species is the thiyl radical. In some cases, the sacrificial additive such as bromotrichloromethane has to be introduced to serve as a direct hydrogen atom transfer (HAT) reagent to activate the thiol for generating a thiyl radical. To avoid the use of sacrificial reagents, Dilman’s group utilized the photoactive Lewis basic species as a photocatalyst [44]. A different reaction pathway, proton coupled electron transfer (PCET), was proposed. The interaction between the thiols and the excited Lewis basic catalyst under blue-light irradiation lead to the formation of thiyl radical for efficient radical thiol-ene reaction to furnish the anti-Markovnikov alkyl sulfides (Scheme 8). This protocol exhibited a broad substrate scope and wide functional group compatibility. Terminal and internal alkenes, aliphatic and aromatic thiols could undergo smoothly in the reaction.

**Scheme 9:**
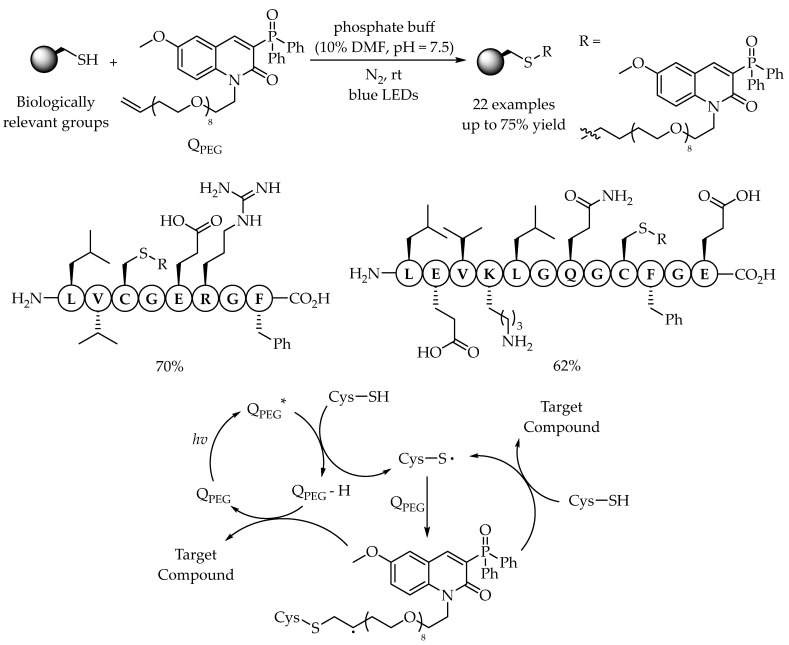
Cysteine-specific bioconjugation by photocatalytic thiol-ene reaction under biocompatible conditions.

As one of the most promising cysteine modification methods, radical thiol-ene reaction for C-S bond formation was still limited because of the need for biocompatible reaction conditions. In 2020, Hong’s group discovered a visible-light-induced biocompatible thiol-ene reaction by using a fluorescent photosensitizer Q_PEG_ for cysteine-specific bioconjugation for the installation of Q_PEG_ [45]. The exclusion of external oxidant and base in this catalytic system inhibited the undesired reaction of peptide and protein substrates with high efficiency and functional group tolerance. Importantly, the fragments of endogenous proteins derived from insulin and Src kinase could be successfully conjugated with Q_PEG_ with the yields of 70% and 62%, respectively. From the mechanistic studies, it was assumed that, under blue LEDs irradiation, the triplet excited state of Q_PEG_ could abstract a hydrogen atom from the cysteine forming a thiyl radical. After the addition of the thiyl radical to the alkene Q_PEG_, an alkyl radical intermediate could be formed, which would abstract a hydrogen atom from another thiol to give rise to the corresponding alkyl sulfide. Alternatively, this alkyl radical could also abstract a hydrogen atom from the intermediate Q_PEG_-H to form the product and regenerate the ground-state Q_PEG_ to close the catalytic cycle (Scheme 9).

**Scheme 10:**
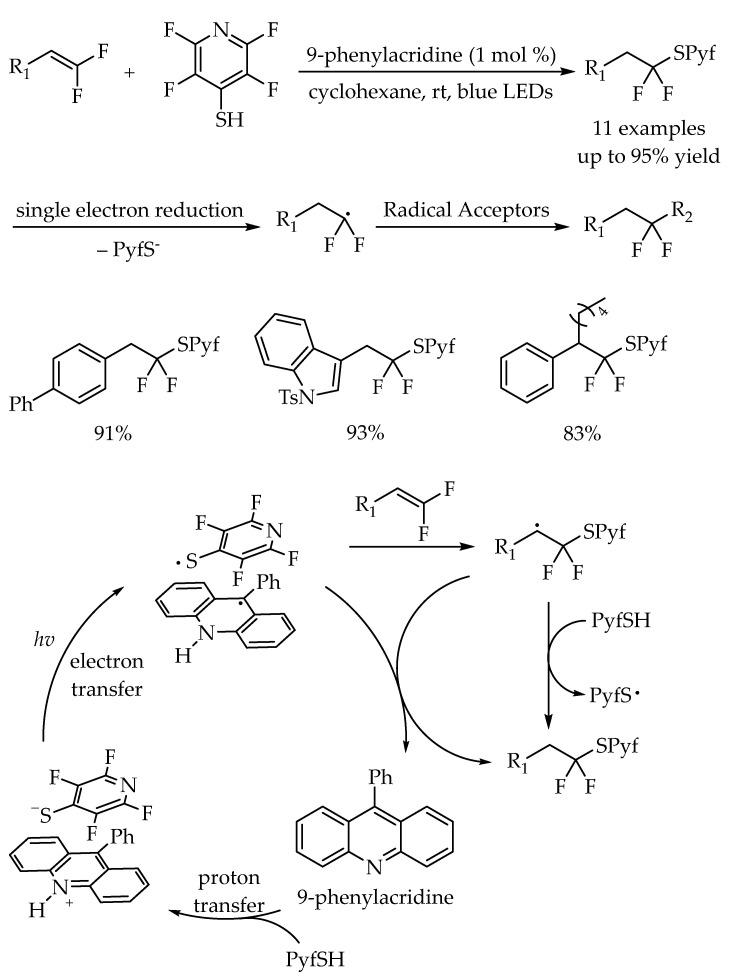
Photocatalyzed thiol-ene reaction for difluorostyrenes functionalization with tetrafluoropyridine-4-thiol.

Recently, the thioethers bearing 4-tetrafluoropyridinylthio and difluoroalkyl groups have been used as precursors to form fluorinated carbon radicals for the construction of difluoroalkylated compounds by Dilman and co-workers [46]. The synthesis of the thioethers involved the photocatalytic thiol-ene reaction using readily accessible difluorostyrenes with tetrafluoropyridine-4-thiol (PyfSH). This protocol was well tolerant of a series of difluoroalkenes bearing aromatic or heteroaromatic groups with good to excellent yields. In addition, a large scale reaction could also undergo smoothly in this protocol. Based on the studies, the authors believed that the generation of thioethers proceeded via a proton coupled electron transfer (PCET) mechanism. The combination of 9-phenylacridine and PyfSH (proton transfer, PT) providing a red colored salt, which could undergo a single electron transfer (SET) process to afford the thiophenol radical under visible light irradiation. After the radical addition to the double bond of difluorostyrenes, the resulting benzyl radical could abstract a hydrogen atom either from the N-H acridinium radical intermediate or from the starting material PyfSH. The measurement of the reduction potential of thioethers indicated that they could participate in a series of reactions with silyl enol ethers, alkenes, nitrones and an alkenyl trifluoroborate (Scheme 10).

### 2.2. Photocatalytic Anti-Markovnikov Thiol-Yne Reactions for Synthesis of Alkenyl Thioethers

**Scheme 11:**
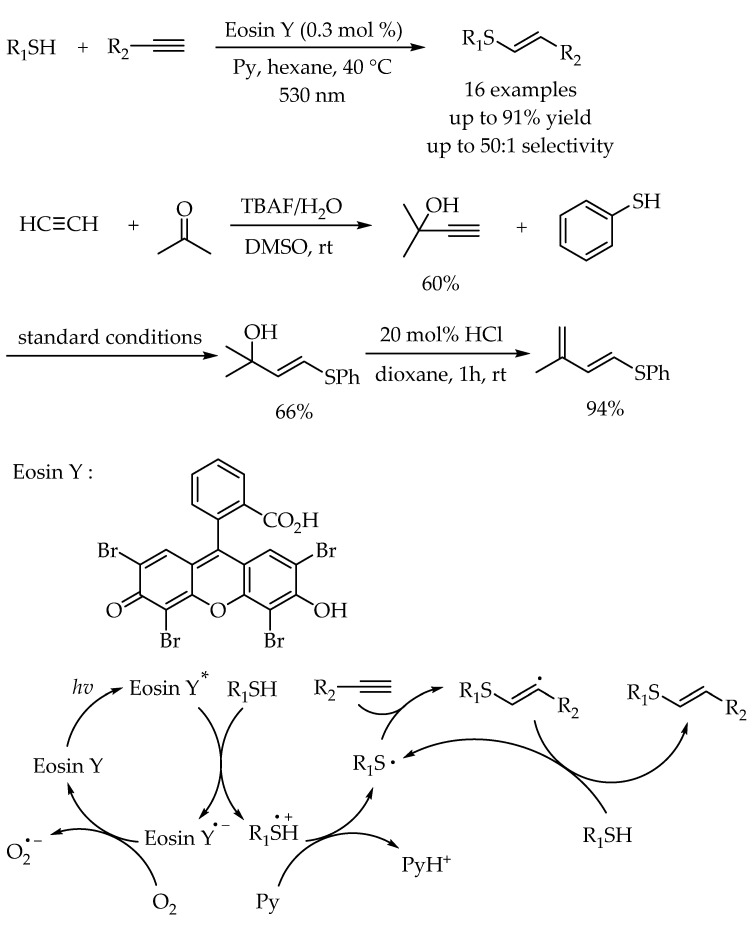
Visible-light-photocatalyzed thiol-yne reaction by Eosin Y.

The addition of thiols to alkynes, namely thiol-yne reaction, is also one of the most direct and atom-economic hydrothiolation reaction to construct carbon-sulfur bonds for synthesis of alkenyl thioethers. However, compared to the synthesis of alkyl sulfides, the examples for synthesis of alkenyl sulfides via photocatalytic thiol-yne reactions were less developed. In 2016, The first example of metal-free photoredox thiol-yne reaction under visible-light irradiation using Eosin Y as a cheap and readily available catalyst was reported by Ananikov and co-workers [47]. Therein, the importance of employing organophotocatalyst is not only to cut the cost, but also to avoid the potential toxic metal contamination. A 3D-printed photoreactor was developed to improve the performance of the catalytic system, and valuable sulfur-functionalized products were obtained in high yields with excellent anti-Markovnikov selectivity. Moreover, the green and efficient synthesis of diene derivatives from industrially available materials demonstrated the potential applicability of this protocol. The reaction mechanism was similar to the previous reported photocatalyzed radical thiol-ene reactions. According to DFT calculations, the high regioselectivity in this reaction was attributed to the fact that the secondary carbon radical generated via anti-Markovnikov addition pathway was more stable than the primary carbon radical (Scheme 11).

**Scheme 12:**
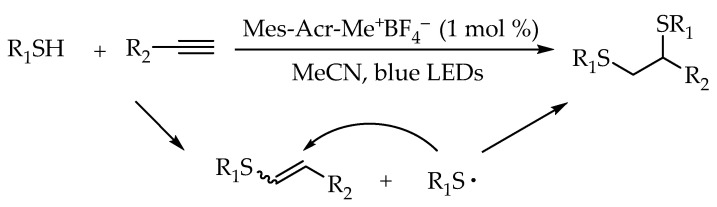
Visible-light-photocatalyzed thiol-yne reaction by 9-mesityl-10-methylacridinium tetrafluoroborate.

In the work reported by Wang’s group in 2019 [48], when less steric hindered thiols were used in the thiol-yne reaction, the difunctionalization of triple bond could happen, and the corresponding alkyl sulfides could be obtained in good yields. However, it was found that the reaction was hard to stay in the first hydrothiolation stage to obtain the alkenyl thioethers. Even though controlling the usage amounts of thiols, the thiol would still react with the generated alkenyl sulfide in this photocatalytic system, leading to a mixture of alkyl sulfides and alkenyl sulfides (Scheme 12).

**Scheme 13:**
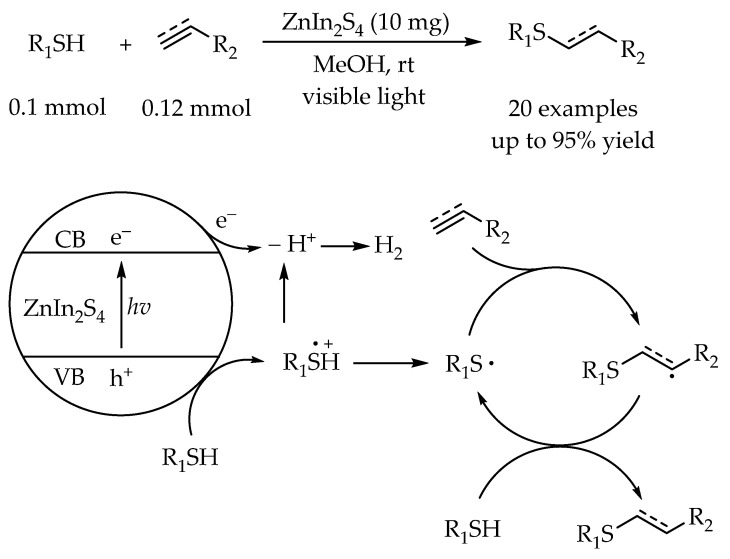
Visible-light-photocatalyzed thiol-ene/yne reaction by ZnIn_2_S_4_.

In the thiol-yne reactions, inorganic semiconductor was also proved to be able to act as a suitable and efficient photocatalyst. Li and co-workers developed a visible-light-initiated hydrothiolation of alkenes and alkynes using ZnIn_2_S_4_ as a photocatalyst in a green solvent [49]. The high functional group tolerance of this reaction was demonstrated by employing various thiols including aromatic thiols and aliphatic thiols. The ESR (electron spin-resonance spectroscopy) and radical inhibition experiments were conducted to elucidate the mechanism. According to the proposed mechanism, the holes generated after photo-excitation of ZnIn_2_S_4_ could efficiently oxidize the thiol to form a thiyl radical, then the anti-Markovnikov addition of the thiyl radical to the alkynes/alkenes would generate the alkenyl/alkyl radical for synthesis of the alkenyl sulfides and alkyl sulfides, respectively (Scheme 13).

### 2.3. Photocatalytic Anti-Markovnikov Thiol-Ene/Yne Reactions for Synthesis of β-Hydroxysulfides/β-Keto Sulfides

**Scheme 14:**
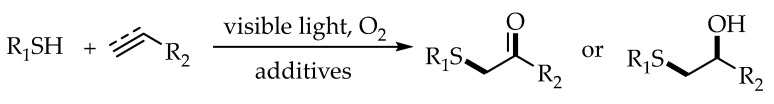
General strategy for synthesis of *β*-hydroxysulfides/*β*-ketone sulfides.

Introducing another functional group during the hydrothiolation reaction at the α or β-position of thioethers was a brilliant strategy to construct more complex thioethers in one-step process. The key point was how to handle the reactive intermediate, the new generated alkenyl/alkyl radical, which was formed via the thiyl radical addition to the unsaturated hydrocarbons. Based on the previous reports, introducing oxygen in the thiol-ene/yne reactions for further C-O bond formation is a good and efficient choice for synthesis of *β*-hydroxysulfides/*β*-keto sulfides (Scheme 14).

**Scheme 15:**
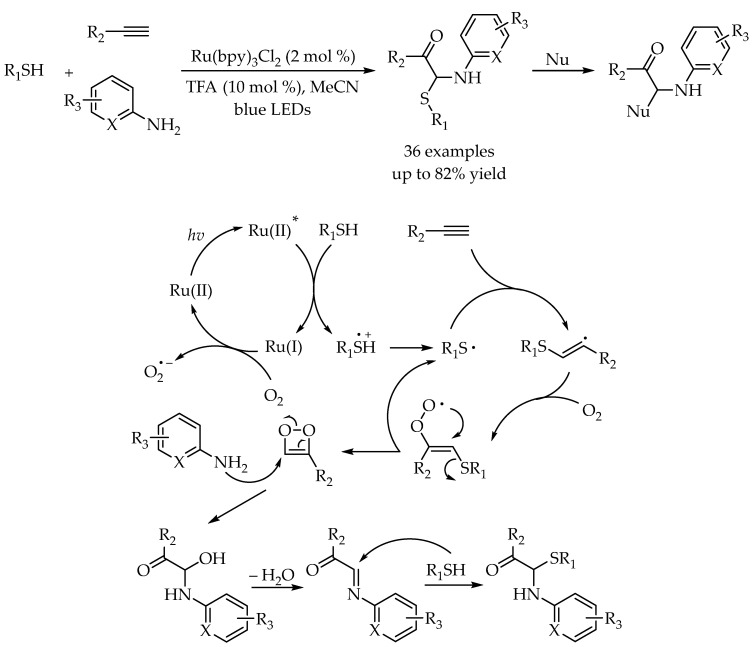
Visible-light-photocatalyzed *gem*-difunctionalization of alkynes for the synthesis of *α,α*-aminothio-substituted carbonyl compounds.

In 2019, Shah and co-workers found that the alkenyl/alkyl radical generated in the radical thiol-yne/ene reaction could undergo oxygen insertion, intermolecular cycloaddition, and nucleophilic addition in the presence of dioxygen and primary arylamine, leading to the construction of α, α-aminothio-substituted carbonyl compounds [50]. The reactions were carried out under mild reaction conditions with broad substrate scope and excellent efficiency. Furthermore, the low bond dissociation energy of the C-S bond in the products has been exploited for selective functionalization using different nucleophiles to build diverse α, α-disubstituted carbonyl scaffolds (Scheme 15).

**Scheme 16:**
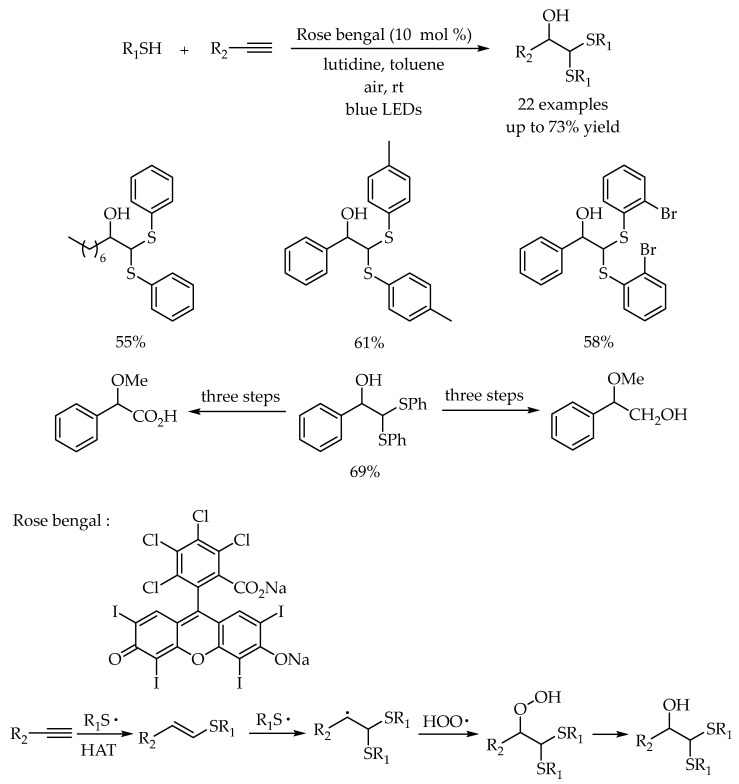
Visible-light photocatalysis for the synthesis of β-hydroxydithioacetals.

Afterward, the same group expanded this strategy to introduce two sulfur functional group at the α-position and a hydroxy group at the β-position in one-step for the synthesis of β-hydroxydithioacetals [51]. Starting from the commercially available terminal alkynes and thiophenols, a series of β-hydroxydithioacetals bearing different functional groups were obtained in moderate yields. Further transformation of the product to useful synthons 2-methoxy-2-phenylacetic acid and 2-methoxy-2-phenylethan-1-ol demonstrated the practicability of this photocatalytic system for valuable organic transformations. The reaction mechanism was proposed to proceed via a radical pathway. The anti-Markovnikov addition of thiyl radical to unsaturated hydrocarbons could occur twice to generate a new carbon radical. Then, the radical cross-coupling of the new carbon radical with the hydroperoxide radical and followed by the reduction of the hydroperoxide intermediate produced the corresponding products (Scheme 16).

**Scheme 17:**
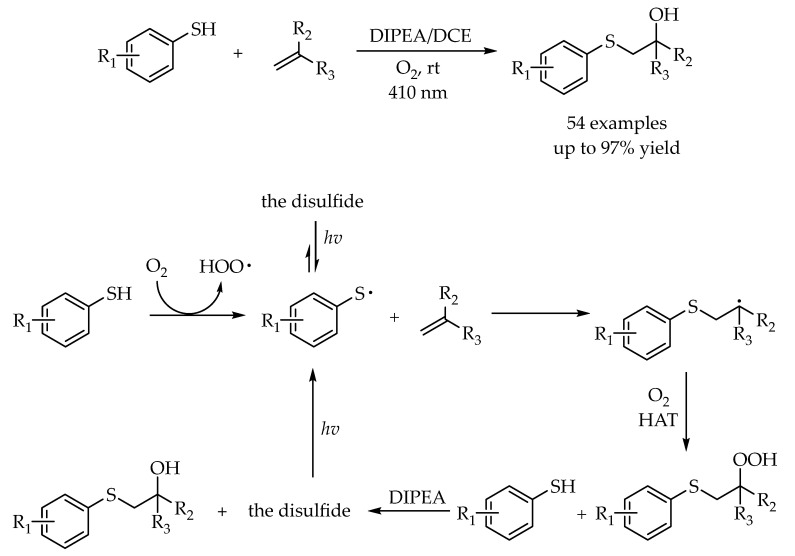
Light-promoted hydroxysulfenylation of alkenes with thiophenols in the presence of tertiary amines.

Recently, our group described a visible-light-promoted and tertiary-amine-assisted strategy for efficient hydroxysulfenylation of alkenes with thiophenols to selectively access β-hydroxysulfides in one-step [52]. Catalyst was unnecessary in this reaction and dioxygen was employed as a green oxidant. Various thiophenols and alkenes could participate in this transformation in good to excellent yields with good functional group tolerance. It is noteworthy that the alkenes with electron-withdrawing groups could also be efficient substrates in this protocol. Moreover, the successful hydroxysulfenylation of structurally complex vinyl-estrone under standard reaction conditions demonstrated that this protocol was a promising strategy for late-stage functionalization of natural products. According to the control experiments and mechanistic investigations, a reasonable reaction pathway was proposed. Under oxygen atmosphere, the thiophenol was first auto-oxidized to generate a thiyl radical, which could attack the alkene to afford an alkyl radical. Then, the generated radical intermediate could quickly react with dioxygen and then followed by a hydrogen atom transfer (HAT) to offer the hydroperoxyl sulfide, which could be in situ reduced directly to the β-hydroxysulfides in the presence of tertiary-amine. Light acting as a driving force played an important role in suppressing the generation of byproduct disulfides and promoted the reaction efficiency (Scheme 17).

### 2.4. Photocatalytic Anti-Markovnikov Thiol–Ene/Yne Reactions for Synthesis of Sulfoxides

**Scheme 18:**
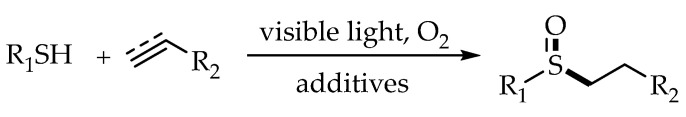
Strategy for synthesis of sulfoxides via thiol–ene/oxidation tandem reaction.

As valuable organic compounds, sulfoxides could be found in many natural and pharmaceutical products. Traditionally, the most direct approach for the synthesis of sulfoxides was the oxidation of thioethers. However, in most cases, two apparent drawbacks were existed in the process. One was the use of excess strong oxidants, which made the reaction usually accompanied with the over oxidation of sulfoxide to sulfone. The other was the multistep operation of the experimental process. Fortunately, the strategy of utilizing tandem thiol-ene reaction cooperated with direct and selective oxidation to sulfoxides was proved to be an efficient alternative (Scheme 18).

**Scheme 19:**
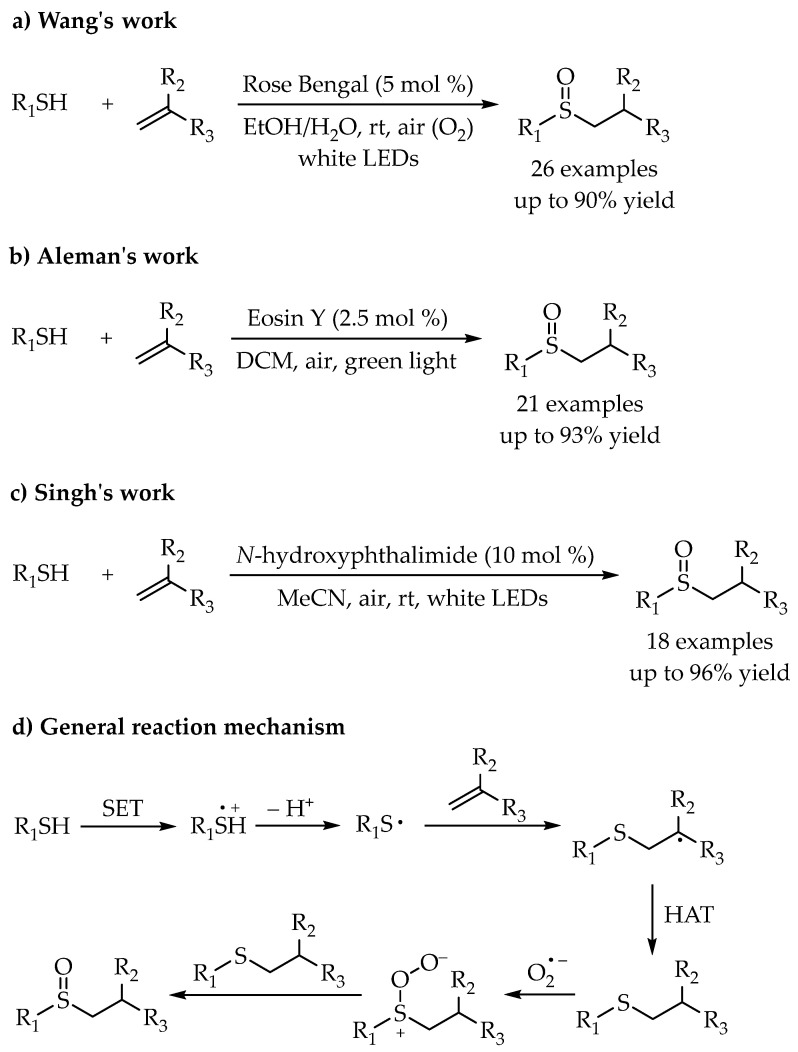
(**a**–**c**) Photocatalyzed selective synthesis of sulfoxides via thiol–ene/yne reaction. (**d**) General reaction mechanism.

In 2017, Wang’s group described a highly selective synthesis of sulfoxides from alkenes and thiols via visible-light photoredox catalysis using air as the environmentally benign oxidant [53]. This protocol was compatible with a broad substrate scope, including aryl thiols, aliphatic thiols and the styrenes bearing electron-rich or -poor groups on the aryl rings (Scheme 19a). Almost at the same time, a similar strategy was reported by Aleman and co-workers [54]. A photocatalytic synthesis of sulfoxides has been carried out using Eosin Y as a catalyst and atmospheric oxygen as the oxidant. Various alkenes and thiols with different functional groups reacted smoothly under the optimized conditions to obtain the corresponding sulfoxides (Scheme 19b). The unrecyclable issue of the catalyst in the above two protocols was unavoidable. To address this problem, Singh’s group developed a stable, inexpensive, more efficient, and reusable photocatalyst NHPI, which was suitable for the thiol-ene/oxidation tandem reaction [55] (Scheme 19c).The mechanism of those reactions was quite similar. Initially, by visible-light irradiation, a single electron transfer (SET) from thiols to the excited photocatalyst provided a radical cation. After deprotonation, the thiyl radical reacted with alkene leading to the formation of alkyl radical, which would undergo a hydrogen atom transfer (HAT) to offer the thioether intermediate. Finally, a direct photooxidation of the thioethers with dioxygen could give the desired sulfoxides (Scheme 19d).

**Scheme 20:**
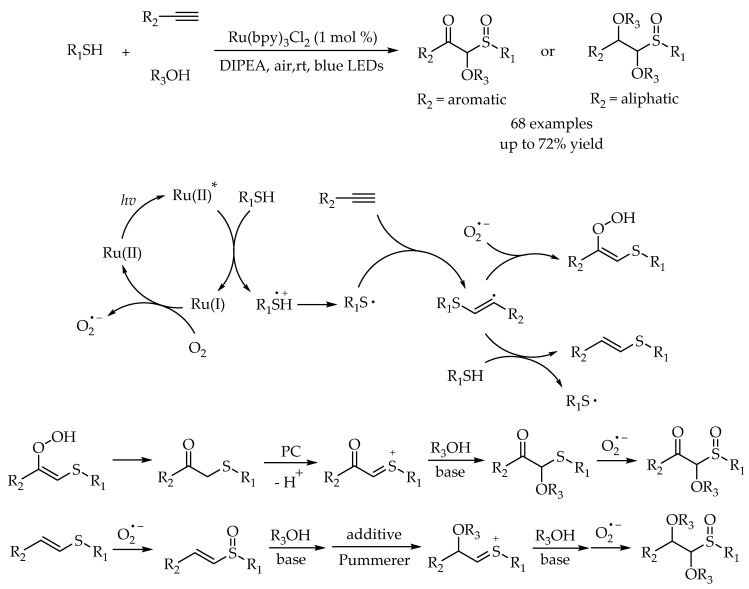
The general mechanism of photoredox synthesis of sulfoxides.

Recently, Shah’s group reported a visible-light photoredox multicomponent reaction for the construction of α-alkoxy-β-ketosulfoxides and α, β-dialkoxysulfoxides using alkynes, thiols, and alcohols as starting materials [56]. This elegant protocol provided a solution to access α-substituted sulfoxides in one-step synthesis. Substrates with various functional groups were available in this transformation, giving rise to the α-substituted sulfoxides in good yields. Based on the control experiments and the previous literatures, a plausible reaction mechanism was proposed. Generally, after the formation of thiyl radical, the radical addition of the thiyl radical to the terminal alkyne could afford the vinyl sulfide intermediate, which could undergo the oxidation and nucleophilic addition continuously to furnish the corresponding products (Scheme 20).

## 3. Photoredox Catalytic Markovnikov-Selective Thiol-Ene/Yne Reactions

As mentioned above, owing to the high reactivity of sulfur, the thiol-ene/yne reaction preferred to proceed via a radical pathway. Hence, it is not surprising that the radical hydrothiolation to access anti-Markovnikov-type products under visible light has been well-developed until now. In contrast, the Markovnikov-selective hydrothiolation reaction still remains a great challenge. Due to the Kharasch effect [57,58] and the catalyst poisoning of metal catalysts by organosulfur compounds, only a few examples of photocatalyzed Markovnikov-selective hydrothiolation reactions have been reported.

### 3.1. Photocatalytic Markovnikov Thiol-Ene Reactions for Synthesis of Branched Alkyl Thioethers

**Scheme 21:**
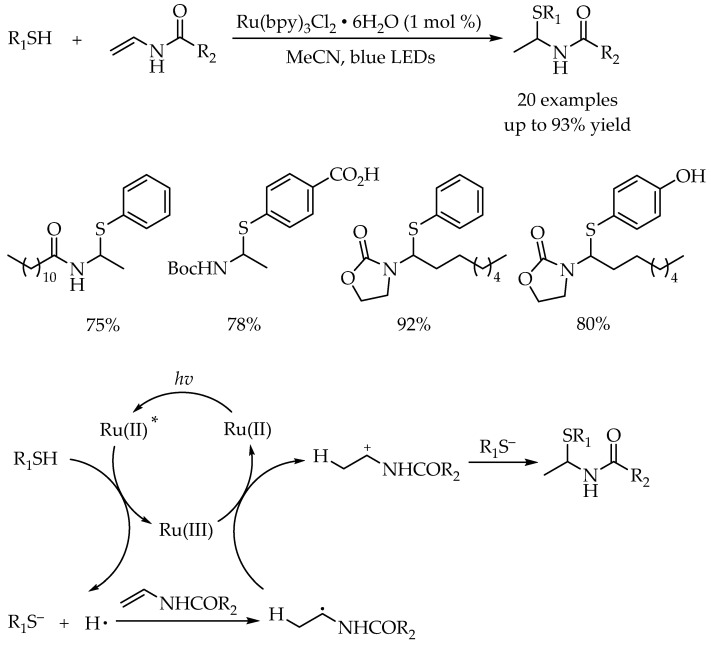
Photocatalyzed Markovnikov-selective hydrothiolation of enamides and enecarbamates.

In 2020, Lim’s group described an efficient visible-light photoredox catalyzed hydrothiolation methodology to regioselectively undergo the functionalization of enamides and enecarbamates to produce the *N*, *S*-acetal products [59]. This reaction exhibited a good substrated scope with broad functional groups. Particularly, when the substrates were disubstituted enamides, excellent yields were still able to be obtained. The mechanism started with the reduction of thiols by the excited photocatalyst, giving a thiolate anion and a hydrogen atom. The hydrogen atom could directly add to the β-position of the double bond on the enamide or enecarbamate to generate an alkyl radical, which could undergo a single electron transfer (SET) with the photocatalyst to be oxidized to form a carbon cation intermediate. Finally, the resulting carbon cation could be directly trapped by the thiolate anion to offer the corresponding Markovnikov-selective product (Scheme 21).

**Scheme 22:**
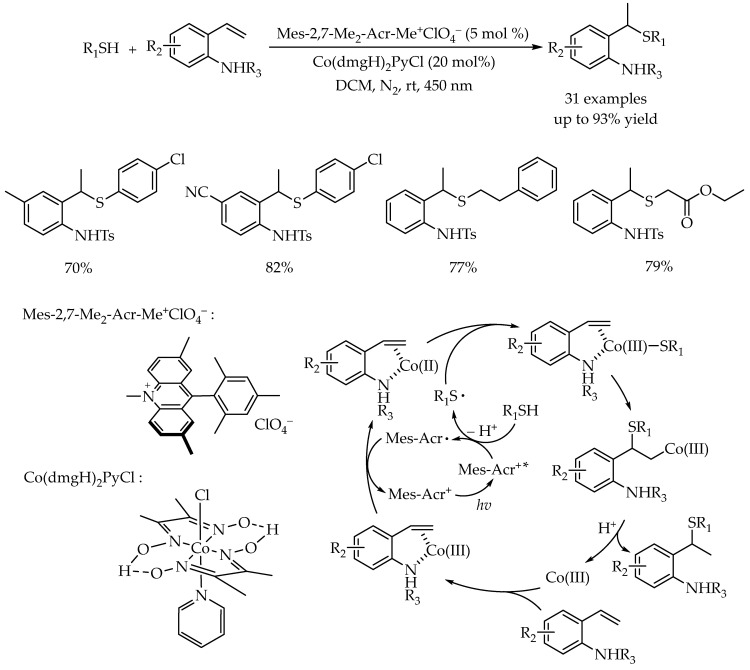
Photoredox/cobalt catalyzed Markovnikov-selective thiol-ene reaction.

One reason for the difficulty of the transition-metal-catalyzed Markovnikov-selective thiol-ene reaction under visible light conditions was the poor coordination ability of alkenes to the transition-metal catalyst. Introducing a directing group to the alkenes was demonstrated to be an excellent solution to address this problem. Recently, our group reported a directing-group-assisted hydrothiolation of styrenes with thiols by photoredox/cobalt catalysis with excellent Markovnikov regioselectivity [60]. This methodology showed broad substrate scope and good functional group tolerance, giving rise to the Markovnikov-type sulfide in good to excellent yields. Substrates bearing electron-rich or -poor groups on the aryl rings provided the corresponding thioethers in satisfactory yields. Moreover, various commercially available alkyl thiols also reacted well with styrenes in this catalytic system. In addition, this reaction could be easily conducted on the gram scale, which demonstrated the practicability of the strategy. On the basis of the mechanism investigations, a reasonable mechanism was proposed. By employing the protected amine group as the directing group, the alkene could be coordinated to a cobalt catalyst to generate the Co(III) species, which was reduced by the radical of photocatalyst, leading to the formation of Co(II) species. Then, the thiyl radical was added to this Co(II) species to produce the cobalt sulfide intermediates. After migration insertion of cobalt sulfide to the alkene with exclusive Markovnikov selectivity, the final protonation would furnish the desired products (Scheme 22).

### 3.2. Photocatalytic Markovnikov Thiol-Yne Reactions for Synthesis of Branched Alkenyl Thioethers

**Scheme 23:**
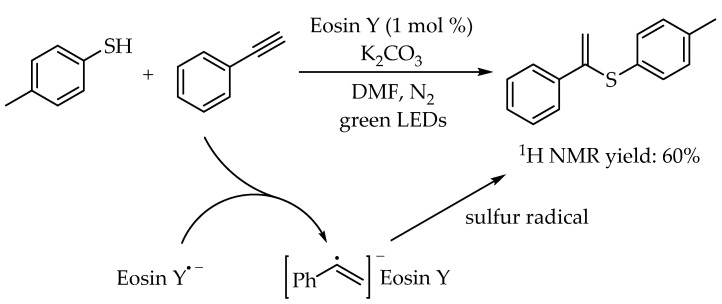
Photocatalytic Markovnikov-selective addition of *p*-toluenethiol to phenylacetylene.

In 2017, Lei and co-workers developed a visible-light-induced radical addition of aryl sulfonic acids to terminal alkynes, giving the α-substituted vinyl sulfones with exclusive Markovnikov regioselectivity [61]. To demonstrate the usefulness of this protocol, the construction of α-substituted vinyl sulfides was also carried out, and the only example provided a 60% ^1^H-NMR yield. For the reaction mechanism, a radical cross-coupling was proposed in this photoredox catalytic system. (Scheme 23).

**Scheme 24:**
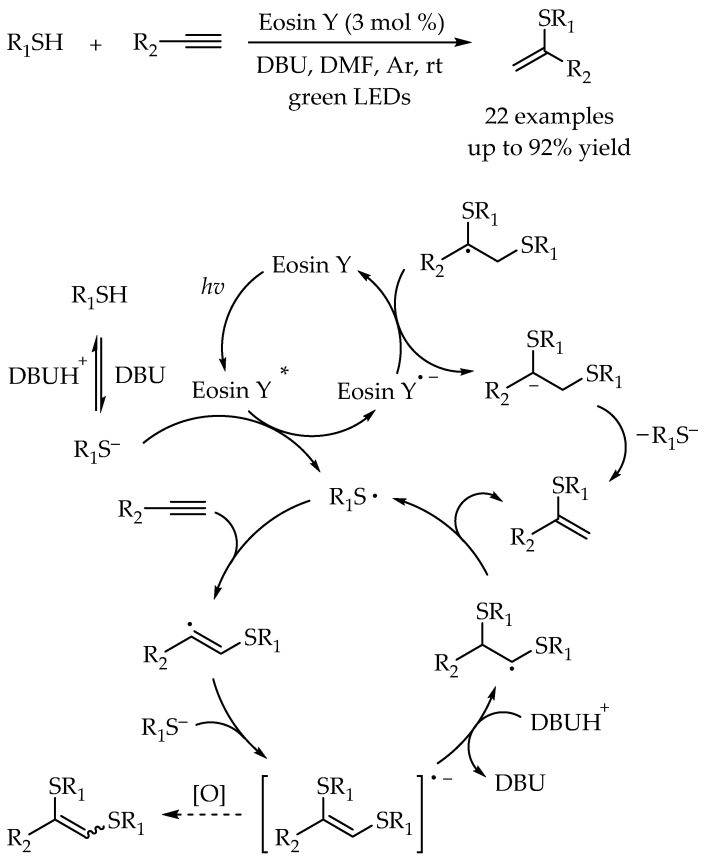
A photocatalyzed Markovnikov thiol-yne reaction via associative electron upconversion.

An associative electron upconversion was proposed to be a key step determining the selectivity of thiol-yne reaction by Ananikov’s group [62]. They developed a transition-metal-free photoredox catalytic system for the selective synthesis of four types of vinyl sulfides, including the challenging Markovnikov-type products. A range of thiophenols and alkynes were well tolerated in this reaction, highlighting the generality of this protocol. A specially designed ESI-MS device was used for directly monitoring the photochemical process and DFT calculations revealed the energy of some crucial intermediates. Based on those reliable data, a possible mechanism was proposed, wherein the thiyl radical was initially formed upon single electron transfer (SET) by photocatalyst. Then, the thiyl radical was added to the alkyne to produce the alkenyl radical, which was able to react with another thiophenol via orbital crossing resulting in the formation of new carbon radical. After being protonated, a stabilized benzyl-type radical could be formed. Subsequent elimination of a thiyl radical yielded the Markovnikov-type product (Scheme 24).

## 4. Conclusions and Outlook

As a powerful tool for the construction of carbon-sulfur bonds, visible-light photoredox catalyzed thiol-ene/yne reactions have been widely explored over the past decade. In this review, recent advances in this rapidly developing area are summarized. In general, the thiol-ene/yne reaction could proceed via a radical pathway or an electrophilic pathway to afford the anti-Markovnikov thioethers or the Markovnikov thioethers, respectively. The research during the past decade mainly focused on the development of various photocatalysts and the investigation of the diversity of the thiol-ene/yne reaction type for green synthesis of more valuable organosulfurs. Owing to the high reactivity of sulfur, the visible-light photoredox catalyzed radical thiol-ene/yne reaction to access various anti-Markovnikov thioethers have been well developed. Despite these achievements in this area, many opportunities and challenges still remain. In many cases of the photocatalytic thiol-ene reactions, a stoichiometric sacrificial reagent such as base, oxidant or radial mediator was needed, which would result in the disadvantage of chemical waste, side reactions and non-atom economy. More simple, efficient catalytic systems are needed to be developed in the future, and the application of these methodologies for the late-stage modifications of bioactive molecules is highly expected. In addition, compared to the anti-Markovnikov-selective thiol-ene/yne reactions, the Markovnikov-selective thiol-ene/yne reactions by photoredox catalysis are still limited, and needed to be further explored to broaden the applications for synthesis of more available organosulfur compounds. Additionally, the combination of photoredox catalysis and metal catalysis may be a promising strategy to promote the development of this intriguing task. Moreover, the photocatalytic asymmetric synthesis of organosulfurs via Markovnikov-selective thiol-ene reaction has not been reported so far. It seems to be a challenging task due to the catalyst poisoning of metal catalysts by sulfur. Even so, the asymmetric organocatalysis is expected to make a contribution to this interesting research field. Hence, we hope that this review will attract more research efforts to this area and provide more insight into the challenging issues in this field to promote the development of visible-light photocatalytic thiol-ene/yne reactions.

## References

[1] Jacob C. (2006). A Scent of Therapy: Pharmacological Implications of Natural Products Containing Redox-Active Sulfur Atoms. Nat. Prod. Rep..

[2] Clayden J., MacLellan P. (2011). Asymmetric Synthesis of Tertiary Thiols and Thioethers. Beilstein J. Org. Chem..

[3] Feng M., Tang B., Liang S.H., Jiang X. (2016). Sulfur Containing Scaffolds in Drugs: Synthesis and Application in Medicinal Chemistry. Curr. Top. Med. Chem..

[4] Wang N., Saidhareddy P., Jiang X. (2020). Construction of Sulfur-Containing Moieties in the Total Synthesis of Natural Products. Nat. Prod. Rep..

[5] Han J., Jia Y., Takeda K., Shiraishi Y., Okamoto M., Dakhama A., Gelfand E.W. (2010). Montelukast during Primary Infection Prevents Airway Hyperresponsiveness and Inflammation after Reinfection with Respiratory Syncytial Virus. Am. J. Respir. Crit. Care Med..

[6] Barré J., Sabatier J.-M., Annweiler C. (2020). Montelukast Drug May Improve COVID-19 Prognosis: A Review of Evidence. Front. Pharmacol..

[7] Qin F., Wang Q., Zhang C., Fang C., Zhang L., Chen H., Zhang M., Cheng F. (2018). Efficacy of Antifungal Drugs in the Treatment of Vulvovaginal Candidiasis: A Bayesian Network Meta-Analysis. Infect. Drug Resist..

[8] Hacioglu M., Guzel C.B., Savage P.B., Tan A.S.B. (2019). Antifungal Susceptibilities, in Vitro Production of Virulence Factors and Activities of Ceragenins against *Candida* Spp. Isolated from Vulvovaginal Candidiasis. Med. Mycol..

[9] Johansson S., Read J., Oliver S., Steinberg M., Li Y., Lisbon E., Mathews D., Leese P.T., Martin P. (2014). Pharmacokinetic Evaluations of the Co-Administrations of Vandetanib and Metformin, Digoxin, Midazolam, Omeprazole or Ranitidine. Clin. Pharmacokinet..

[10] McGarrigle E.M., Myers E.L., Illa O., Shaw M.A., Riches S.L., Aggarwal V.K. (2007). Chalcogenides as Organocatalysts. Chem. Rev..

[11] Hoyle C.E., Lowe A.B., Bowman C.N. (2010). Thiol-Click Chemistry: A Multifaceted Toolbox for Small Molecule and Polymer Synthesis. Chem. Soc. Rev..

[12] Arrayás R.G., Carretero J.C. (2011). Chiral Thioether-Based Catalysts in Asymmetric Synthesis: Recent Advances. Chem. Commun..

[13] Yuan D., Huynh H.V. (2012). Sulfur-Functionalized N-Heterocyclic Carbene Complexes of Pd(II): Syntheses, Structures and Catalytic Activities. Molecules.

[14] Lynch D.M., Scanlan E.M. (2020). Thiyl Radicals: Versatile Reactive Intermediates for Cyclization of Unsaturated Substrates. Molecules.

[15] Gress A., Völkel A., Schlaad H. (2007). Thio-Click Modification of Poly[2-(3-Butenyl)-2-Oxazoline]. Macromolecules.

[16] ten Brummelhuis N., Diehl C., Schlaad H. (2008). Thiol−Ene Modification of 1,2-Polybutadiene Using UV Light or Sunlight. Macromolecules.

[17] Hoyle C.E., Bowman C.N. (2010). Thiol-Ene Click Chemistry. Angew. Chem. Int. Ed..

[18] Scanlan E., Corcé V., Malone A. (2014). Synthetic Applications of Intramolecular Thiol-Ene “Click” Reactions. Molecules.

[19] Sánchez-Fernández E.M., García-Moreno M.I., García-Hernández R., Padrón J.M., García Fernández J.M., Gamarro F., Ortiz Mellet C. (2019). Thiol-Ene “Click” Synthesis and Pharmacological Evaluation of C-Glycoside Sp^2^-Iminosugar Glycolipids. Molecules.

[20] Hearon K., Nash L.D., Rodriguez J.N., Lonnecker A.T., Raymond J.E., Wilson T.S., Wooley K.L., Maitland D.J. (2014). A High-Performance Recycling Solution for Polystyrene Achieved by the Synthesis of Renewable Poly(Thioether) Networks Derived from D-Limonene. Adv. Mater..

[21] Kumar R., Saima, Shard A., Andhare N.H., Richa, Sinha A.K. (2015). Thiol-Ene “Click” Reaction Triggered by Neutral Ionic Liquid: The “Ambiphilic” Character of [Hmim]Br in the Regioselective Nucleophilic Hydrothiolation. Angew. Chem. Int. Ed..

[22] Healy J., Rasmussen T., Miller S., Booth I.R., Conway S.J. (2016). The Photochemical Thiol–Ene Reaction as a Versatile Method for the Synthesis of Glutathione S-Conjugates Targeting the Bacterial Potassium Efflux System Kef. Org. Chem. Front..

[23] Vanslambrouck S., Riva R., Ucakar B., Préat V., Gagliardi M., Molin D.G.M., Lecomte P., Jérôme C. (2021). Thiol-Ene Reaction: An Efficient Tool to Design Lipophilic Polyphosphoesters for Drug Delivery Systems. Molecules.

[24] Shi L., Xia W. (2012). Photoredox Functionalization of C–H Bonds Adjacent to a Nitrogen Atom. Chem. Soc. Rev..

[25] Prier C.K., Rankic D.A., MacMillan D.W.C. (2013). Visible Light Photoredox Catalysis with Transition Metal Complexes: Applications in Organic Synthesis. Chem. Rev..

[26] Ghosh I., Marzo L., Das A., Shaikh R., König B. (2016). Visible Light Mediated Photoredox Catalytic Arylation Reactions. Acc. Chem. Res..

[27] Kärkäs M.D., Porco J.A., Stephenson C.R.J. (2016). Photochemical Approaches to Complex Chemotypes: Applications in Natural Product Synthesis. Chem. Rev..

[28] Skubi K.L., Blum T.R., Yoon T.P. (2016). Dual Catalysis Strategies in Photochemical Synthesis. Chem. Rev..

[29] Chen B., Wu L.-Z., Tung C.-H. (2018). Photocatalytic Activation of Less Reactive Bonds and Their Functionalization via Hydrogen-Evolution Cross-Couplings. Acc. Chem. Res..

[30] Zhong J.-J., To W.-P., Liu Y., Lu W., Che C.-M. (2019). Efficient Acceptorless Photo-Dehydrogenation of Alcohols and N-Heterocycles with Binuclear Platinum(II) Diphosphite Complexes. Chem. Sci..

[31] Qi X.-K., Zhang H., Pan Z.-T., Liang R.-B., Zhu C.-M., Li J.-H., Tong Q.-X., Gao X.-W., Wu L.-Z., Zhong J.-J. (2019). Photoinduced Synthesis of Fluorinated Dibenz[*b*,*e*]Azepines via Radical Triggered Cyclization. Chem. Commun..

[32] Zhang H., Xiao Q., Qi X.-K., Gao X.-W., Tong Q.-X., Zhong J.-J. (2020). Selective Photoredox Decarboxylation of *α*-Ketoacids to Allylic Ketones and 1,4-Dicarbonyl Compounds Dependent on Cobaloxime Catalysis. Chem. Commun..

[33] Xu H., Zhang H., Tong Q.-X., Zhong J.-J. (2021). Photoredox/Cobaloxime Co-Catalyzed Allylation of Amines and Sulfonyl Hydrazines with Olefins to Access *α*-Allylic Amines and Allylic Sulfones. Org. Biomol. Chem..

[34] Lu F.-D., Chen J., Jiang X., Chen J.-R., Lu L.-Q., Xiao W.-J. (2021). Recent Advances in Transition-Metal-Catalysed Asymmetric Coupling Reactions with Light Intervention. Chem. Soc. Rev..

[35] Xiao Q., Lu M., Deng Y., Jian J.-X., Tong Q.-X., Zhong J.-J. (2021). Photoinduced Radical Cascade Cyclization: A Metal-Free Approach to Access Difluoroalkylated Dioxodibenzothiazepines. Org. Lett..

[36] Tyson E.L., Ament M.S., Yoon T.P. (2013). Transition Metal Photoredox Catalysis of Radical Thiol-Ene Reactions. J. Org. Chem..

[37] Keylor M.H., Park J.E., Wallentin C.-J., Stephenson C.R.J. (2014). Photocatalytic Initiation of Thiol–Ene Reactions: Synthesis of Thiomorpholin-3-Ones. Tetrahedron.

[38] Bhat V.T., Duspara P.A., Seo S., Abu Bakar N.S.B., Greaney M.F. (2015). Visible Light Promoted Thiol-Ene Reactions Using Titanium Dioxide. Chem. Commun..

[39] Fadeyi O.O., Mousseau J.J., Feng Y., Allais C., Nuhant P., Chen M.Z., Pierce B., Robinson R. (2015). Visible-Light-Driven Photocatalytic Initiation of Radical Thiol–Ene Reactions Using Bismuth Oxide. Org. Lett..

[40] Limnios D., Kokotos C.G. (2017). Photoinitiated Thiol-Ene “Click” Reaction: An Organocatalytic Alternative. Adv. Synth. Catal..

[41] Singh M., Yadav A.K., Yadav L.D.S., Singh R.K.P. (2017). Visible Light Photocatalysis with Benzophenone for Radical Thiol-Ene Reactions. Tetrahedron Lett..

[42] Zhao G., Kaur S., Wang T. (2017). Visible-Light-Mediated Thiol–Ene Reactions through Organic Photoredox Catalysis. Org. Lett..

[43] Liu H., Chung H. (2017). Visible-Light Induced Thiol–Ene Reaction on Natural Lignin. ACS Sustain. Chem. Eng..

[44] Levin V.V., Dilman A.D. (2019). Visible-Light-Mediated Organocatalyzed Thiol–Ene Reaction Initiated by a Proton-Coupled Electron Transfer. J. Org. Chem..

[45] Choi H., Kim M., Jang J., Hong S. (2020). Visible-Light-Induced Cysteine-Specific Bioconjugation: Biocompatible Thiol–Ene Click Chemistry. Angew. Chem. Int. Ed..

[46] Zubkov M.O., Kosobokov M.D., Levin V.V., Kokorekin V.A., Korlyukov A.A., Hu J., Dilman A.D. (2020). A Novel Photoredox-Active Group for the Generation of Fluorinated Radicals from Difluorostyrenes. Chem. Sci..

[47] Zalesskiy S.S., Shlapakov N.S., Ananikov V.P. (2016). Visible Light Mediated Metal-Free Thiol–Yne Click Reaction. Chem. Sci..

[48] Kaur S., Zhao G., Busch E., Wang T. (2019). Metal-Free Photocatalytic Thiol–Ene/Thiol–Yne Reactions. Org. Biomol. Chem..

[49] Li Y., Cai J., Hao M., Li Z. (2019). Visible Light Initiated Hydrothiolation of Alkenes and Alkynes over ZnIn2S4. Green Chem..

[50] Chalotra N., Rizvi M.A., Shah B.A. (2019). Photoredox-Mediated Generation of Gem -Difunctionalized Ketones: Synthesis of *α*,*α*-Aminothioketones. Org. Lett..

[51] Manzer Manhas F., Kumar J., Raheem S., Thakur P., Rizvi M.A., Shah B.A. (2021). Photoredox-Mediated Synthesis of β-Hydroxydithioacetals from Terminal Alkynes. ChemPhotoChem.

[52] Shi J., Gao X.-W., Tong Q.-X., Zhong J.-J. (2021). Light-Promoted and Tertiary-Amine-Assisted Hydroxysulfenylation of Alkenes: Selective and Direct One-Pot Synthesis of β-Hydroxysulfides. J. Org. Chem..

[53] Cui H., Wei W., Yang D., Zhang Y., Zhao H., Wang L., Wang H. (2017). Visible-Light-Induced Selective Synthesis of Sulfoxides from Alkenes and Thiols Using Air as the Oxidant. Green Chem..

[54] Guerrero-Corella A., María Martinez-Gualda A., Ahmadi F., Ming E., Fraile A., Alemán J. (2017). Thiol–Ene/Oxidation Tandem Reaction under Visible Light Photocatalysis: Synthesis of Alkyl Sulfoxides. Chem. Commun..

[55] Singh M., Yadav A.K., Yadav L.D.S., Singh R.K.P. (2018). Visible-Light-Activated Selective Synthesis of Sulfoxides via Thiol-Ene/Oxidation Reaction Cascade. Tetrahedron Lett..

[56] Kumar J., Ahmad A., Rizvi M.A., Ganie M.A., Khajuria C., Shah B.A. (2020). Photoredox-Mediated Synthesis of Functionalized Sulfoxides from Terminal Alkynes. Org. Lett..

[57] Gossage R.A., van de Kuil L.A., van Koten G. (1998). Diaminoarylnickel(II) “Pincer” Complexes: Mechanistic Considerations in the Kharasch Addition Reaction, Controlled Polymerization, and Dendrimeric Transition Metal Catalysts. Acc. Chem. Res..

[58] Wille U. (2013). Radical Cascades Initiated by Intermolecular Radical Addition to Alkynes and Related Triple Bond Systems. Chem. Rev..

[59] Barman E., Hourizadeh J., Lim D. (2020). Visible Light Photoredox-Catalyzed Hydrothiolation of Enamides and Enecarbamates. Tetrahedron Lett..

[60] Xiao Q., Zhang H., Li J.-H., Jian J.-X., Tong Q.-X., Zhong J.-J. (2021). Directing-Group-Assisted Markovnikov-Selective Hydrothiolation of Styrenes with Thiols by Photoredox/Cobalt Catalysis. Org. Lett..

[61] Wang H., Lu Q., Chiang C.-W., Luo Y., Zhou J., Wang G., Lei A. (2017). Markovnikov-Selective Radical Addition of S-Nucleophiles to Terminal Alkynes through a Photoredox Process. Angew. Chem. Int. Ed..

[62] Burykina J.V., Shlapakov N.S., Gordeev E.G., König B., Ananikov V.P. (2020). Selectivity Control in Thiol–Yne Click Reactions via Visible Light Induced Associative Electron Upconversion. Chem. Sci..

